# Association between disease activity of rheumatoid arthritis and risk of complications following total hip arthroplasty: a retrospective cohort study

**DOI:** 10.1186/s13018-024-04924-4

**Published:** 2024-07-31

**Authors:** Yahao Lai, Haiwei Tang, Zichuan Ding, Chao Huang, Yongrui Cai, Zeyu Luo, Zongke Zhou

**Affiliations:** Department of Orthopaedic Surgery, West China Hospital of Sichuan University, No.37, Guoxue Road, Wuhou district, Chengdu, Sichuan province 610041 China

**Keywords:** Rheumatoid arthritis, Activity, Complication, Total hip arthroplasty

## Abstract

**Background:**

Identifying rheumatoid arthritis patients at higher risk of complications after total hip arthroplasty could make perioperative management more effective. Here we examined whether disease activity is associated with risk of such complications.

**Methods:**

We retrospectively analyzed data for 337 rheumatoid arthritis patients at our medical center who underwent primary total hip arthroplasty. Rheumatoid arthritis patients were categorized according to the simplified disease activity index (SDAI), the values of which at admission and follow-up were averaged together. Logistic regression was used to examine associations of mean SDAI with rates of dislocation, infection, periprosthetic fracture and aseptic loosening. As controls, 337 osteoarthritis patients who did not have systemic inflammation and who underwent the same procedure were matched across numerous clinicodemographic variables.

**Results:**

Among the 337 rheumatoid arthritis patients, 38 (11.3%) had postoperative complications, the rates of which varied significantly from 0 to 17.5% (*p* = 0.003) among the four subgroups whose disease activity based on mean SDAI was categorized as high, moderate, low or in remission. Each 1-unit increase in mean SDAI was associated with a significant increase in risk of postoperative complications (OR 1.015, 95% CI 1.001–1.029, *p* = 0.035). Across all rheumatoid arthritis patients, rate of complications did not differ significantly between patients who received disease-modifying anti-rheumatic drugs or other treatments. Rates of dislocation, of infection or of all postoperative complications combined were significantly lower among osteoarthritis controls than among rheumatoid arthritis patients.

**Conclusion:**

Greater mean SDAI is associated with higher risk of dislocation, infection and composite postoperative complications after total hip arthroplasty in rheumatoid arthritis patients. These patients show a significantly higher rate of postoperative complications than osteoarthritis patients, likely reflecting the influence of systemic inflammation. Disease activity should be reduced as much as possible in rheumatoid arthritis patients before they undergo total hip arthroplasty.

## Background

Rheumatoid arthritis is a chronic, progressive autoimmune inflammatory disease that primarily affects joints and is characterized by aggressive, polyarticular, symmetric inflammation of the small joints in the hands and feet, and it can involve tissues outside the joints [1]. The incidence of rheumatoid arthritis, which has been increasing for the past 30 years, is projected to reach 10.48 per 100 000 women and 4.63 per 100 000 men by 2040 [2]. Even with improvements in drug therapies, approximately one quarter of rheumatoid arthritis patients require total joint arthroplasty [3]. Among those patients who undergo surgery, up to 5.7% experience postoperative complications, such as dislocation, infection and periprosthetic fracture [4]. Identifying which patients are at higher risk of such complications may improve perioperative management and channel healthcare resources efficiently.

Since the systemic inflammation in rheumatoid arthritis can drive osteolysis and injury of periarticular soft tissues and has been linked to higher rates of complications after total joint arthroplasty [5], we wondered whether disease activity might predict risk of postoperative complications. To test this possibility, we compared rates of complications between rheumatoid arthritis patients showing weaker or stronger disease activity who underwent total hip arthroplasty at our medical center. As an additional exploration of the potential contribution of systemic inflammation to postoperative complications, we compared rates of complications between the rheumatoid arthritis patients and a matched group of osteoarthritis patients, who do not show systemic inflammation [6]. Given the potential of some drug therapies to affect immune responses and therefore risk of postoperative infection [7], we also compared complication rates between rheumatoid arthritis patients who received different types of drugs.

## Methods

We retrospectively analyzed medical records of patients with rheumatoid arthritis or osteoarthritis who underwent primary total hip arthroplasty at our medical center between January 2010 and June 2022. Our study was designed in accordance with the Declaration of Helsinki and was approved by the Ethics Committee on Biomedical Research of our medical center (approval 2023 − 2005), which waived the need for written informed consent because patients or their legal guardians, at the time of admission, signed written consent for patients’ anonymized data to be analyzed and published for research purposes.

### Rheumatoid arthritis patients

**Fig. 1:**
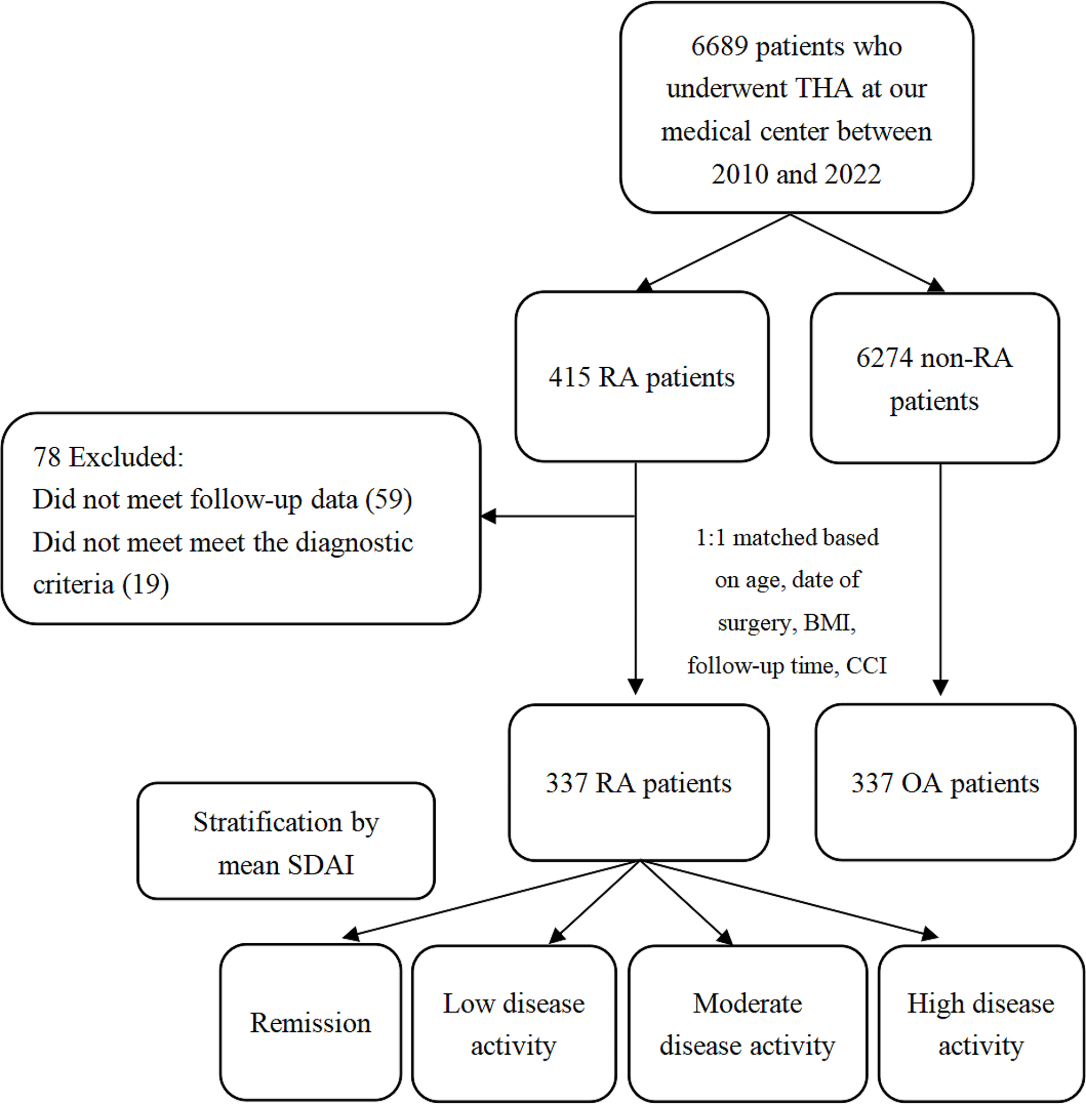
Flowchart showing patient selection, stratification, and matching. BMI, body mass index; CCI, Charlson comorbidity index; OA, osteoarthritis; RA, rheumatoid arthritis; SDAI, simplified disease activity index; THA, total hip arthroplasty

We analyzed a consecutive series of patients at least 18 years old but no older than 80 years who underwent total hip arthroplasty at our center between January 2010 and June 2022, who fulfilled the diagnostic criteria of rheumatoid arthritis of the American College of Rheumatology and European League Against Rheumatism [8], and for whom follow-up data were available. Patients were classified as being in remission or having low, moderate, or high disease activity based on the average of the simplified disease activity index (SDAI) [9] at admission and the SDAI after surgery at each follow-up (Fig. 1). Remission was defined as mean SDAI ≤ 3.3; low disease activity, 3.3 < mean SDAI ≤ 11.0; moderate disease activity, 11.0 < mean SDAI ≤ 26; and high disease activity, mean SDAI > 26 [10]. The four groups of patients did not differ significantly in any clinicodemographic variable that we examined.

### Osteoarthritis patients

We analyzed osteoarthritis patients who were matched 1:1 to the rheumatoid arthritis patients on the basis of age (within 5 years), date of surgery (within 1 year), body mass index (within the same category), follow-up time (within 1 year), and Charlson comorbidity index [11] (within 1 point). The following categories of body mass index were defined [12]: underweight, < 18.5 kg/m^2^; normal weight, 18.5–24.9 kg/m^2^; overweight, 25.0–29.9 kg/m^2^; obesity, ≥ 30.0 kg/m^2^. The two groups of patients did not differ significantly in any clinicodemographic variable that we examined.

### Outcomes

Rates of postoperative complications were recorded, including dislocation of the prosthesis, periprosthetic joint infection, superficial infection, periprosthetic fracture, and aseptic loosening. Aseptic loosening was defined as a radiolucent line at least 2 mm wide; non-linear regions of intra-osseous, cortical, or cancellous bone destruction at least 2 mm thick; or migration > 2 mm of prosthesis components.

### Follow-up

Patients were followed up annually on an outpatient basis, when clinical and laboratory tests were performed as well as x-ray imaging of the prosthesis. All enrolled patients completed at least two times follow-up. At each follow-up, the activity was determined in order to calculate the SDAI. Patients were told to go immediately to the emergency department in the event of abnormal hip pain, poor skin healing, or surgical incision infection. Patients who reported abnormal pain were asked to undergo pelvic anteroposterior X-ray imaging to rule out dislocation of their prosthesis. All patients who were suspected of having infection were assessed by an experienced joint surgeon; in these cases, secretions were cultured for bacteria, or hip puncture was performed. At the last follow-up, the radiographs were read by an experienced joint surgeon.

### Data analysis

Continuous data were reported as mean ± SD or median (interquartile range), while categorical data were reported as n (%). Intergroup differences in categorical variables were assessed for significance using the Pearson’s chi-squared test. Differences in rates of complications between patients with different levels of disease activity were assessed using the chi-squared test. Differences in rates of complications between rheumatoid arthritis patients and osteoarthritis patients were assessed using the chi-squared test. Logistic regression was used to explore potential associations of mean SDAI or type of drug treatment with postoperative complications in rheumatoid arthritis patients. When appropriate, results were reported in terms of odds ratios (ORs) and associated 95% confidence intervals (CIs). All analyses were carried out using SPSS 26 (IBM, Armonk, NY, USA). Significance was defined as *p* < 0.05.

## Results

**Fig. 2:**
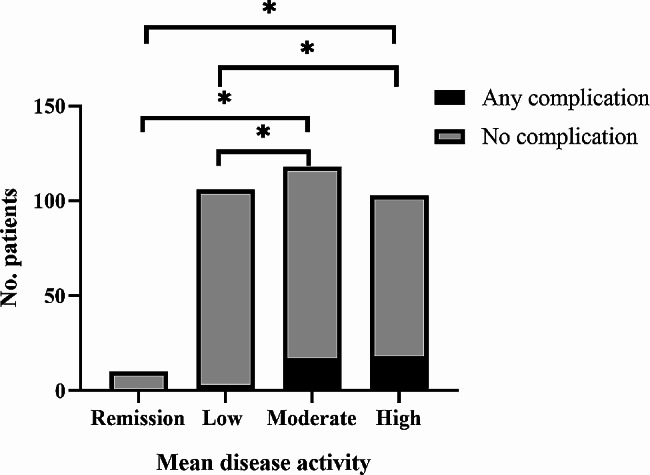
Numbers of rheumatoid arthritis patients, stratified by the mean value of the simplified disease activity index at admission and follow-up, who experienced any or no complications after total hip arthroplasty. *, *p* < 0.05

**Fig. 3:**
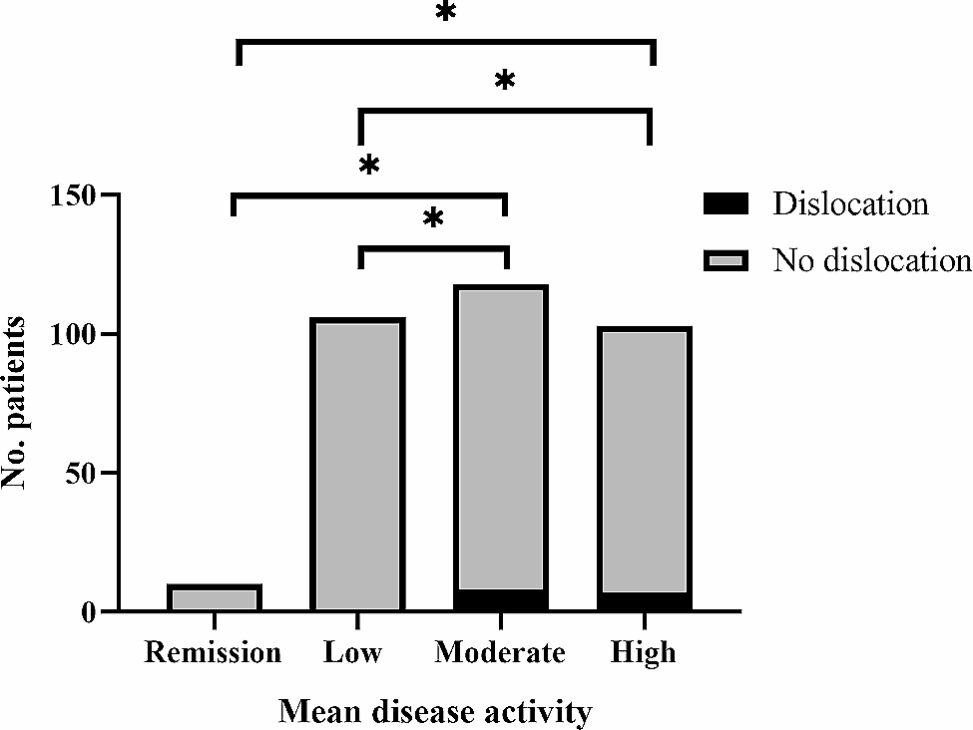
Numbers of rheumatoid arthritis patients, stratified by the mean value of the simplified disease activity index at admission and follow-up, who experienced dislocation or not after total hip arthroplasty. *, *p* < 0.05

**Fig. 4:**
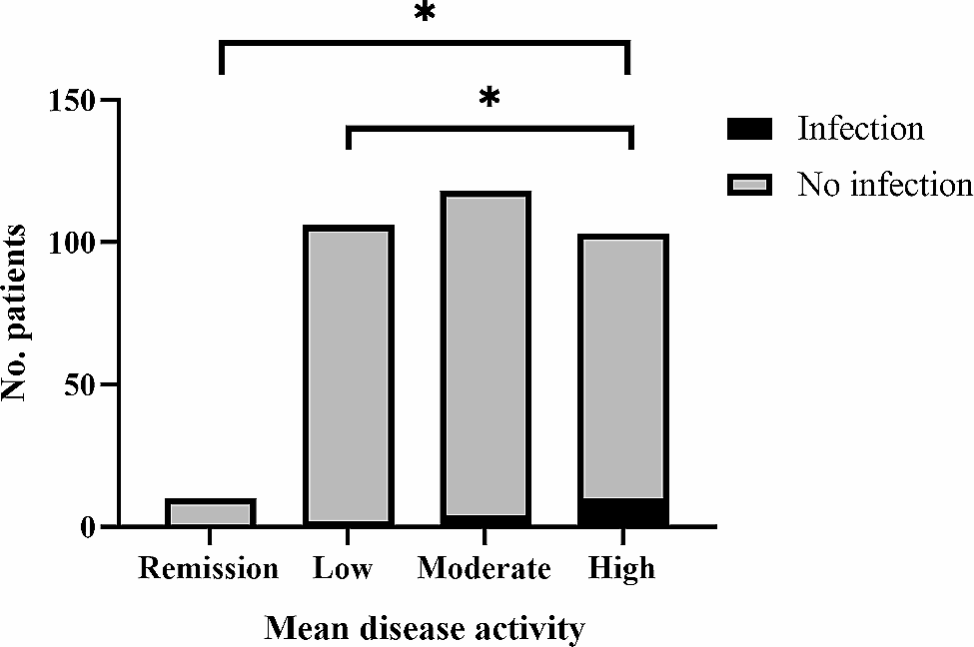
Numbers of rheumatoid arthritis patients, stratified by the mean value of the simplified disease activity index, who experienced surgical site infection or not after total hip arthroplasty. *, *p* < 0.05

Of 415 rheumatoid arthritis patients who underwent total hip arthroplasty during our enrollment period, we included 337 who fulfilled the inclusion criteria, based on whom we included a matched control group of osteoarthritis patients who underwent the same surgery.The mean SDAI scores of the four groups were 2.7 ± 0.3, 9.0 ± 1.3, 16.8 ± 4.2, and 54.2 ± 16.7, respectively (Table 1). Additionally, we observed that 64 out of 104 patients (61.5%) who were administered glucocorticoids prior to surgery experienced a reduction in their glucocorticoid dosage post-surgery. Among the 97 patients receiving DMARDs, 22 individuals (22.7%) exhibited a decrease in their medication dosage. Of 97 cases of patients using DMARDs, 22 cases (22.7%) patients with reduced dose of drug. Among rheumatoid arthritis patients, 38 (11.3%) suffered postoperative complications, and the rate varied significantly from 0 to 17.5% (*p* = 0.003) among the four subgroups who were in remission or who had low, moderate or high disease activity. Among individual complications, the four groups differed significantly in incidence of hip dislocation (*p* = 0.041) and infection (*p* = 0.037) (Table 2). Logistic regression linked each 1-unit increase in mean SDAI to significantly higher risk of postoperative complications (OR 1.015, 95% CI 1.001–1.029, *p* = 0.035) (Table 3). In *post hoc* comparisons among the four disease activity subgroups, those who showed moderate or high activity were at significantly higher risk of postoperative complications in general and of dislocation in particular than the two other groups (Figs. 2 and 3). Patients who showed high activity were at significantly higher risk of infection than those who were in remission or showed low activity (Fig. 4).

**Table 1:** Clinicodemographic characteristics of rheumatoid arthritis patients and osteoarthritis patients in this study

Characteristic	Rheumatoid arthritis subgroups by mean disease activity*				*p*	Rheumatoid arthritis patients (*N* = 337)	Osteoarthritis patients (*N* = 337)	*p*
	Remission (*n* = 10)	Low (*n* = 106)	Moderate (*n* = 118)	High (*n* = 103)				
Sex					0.771			0.105
Male	3 (30)	24 (22.6)	22 (18.6)	23 (22.3)		72 (21.4)	90 (26.7)	
Female	7 (70)	82 (77.4)	96 (81.4)	80 77.7)		265 (78.6)	247 (73.3)	
Age, yr	49.8 ± 12.1	50.7 ± 13.2	51.4 ± 12.2	52.7 ± 13.1	0.657	51.5 ± 12.8	51.9 ± 12.6	0.765
Body mass index, kg/m^2^	20.9 ± 2.8	21.9 ± 3.4	22.0 ± 3.4	22.4 ± 3.7	0.449	22.1 ± 3.5	22.6 ± 4.1	0.089
Hip side					0.657			0.355
Right	6 (60)	52 (49.1)	52 (44.1)	52 (50.5)		160 (47.5)	172 (51.0)	
Left	4 (40)	54 (50.9)	66 (55.9)	51 (49.5)		177 (52.5)	165 (49.0)	
Drugs								
Glucocorticoids	3 (30)	33 (31.1)	33 (28.0)	35 (34.0)	0.816	104 (30.9)	-	
DMARDs	1 (10)	31 (29.2)	32 (27.1)	33 (32.0)	0.489	97 (28.8)	-	
Charlson comorbidity index					0.991			0.127
< 2	6 (60)	66 (62.3)	71 (60.2)	63 (61.2)		206 (61.1)	225 (66.8)	
≥ 2	4 (40)	40 (37.7)	47 (39.8)	40 (38.8)		131 (38.9)	112 (33.2)	
Mean SDAI	2.7 ± 0.3	9.1 ± 1.3	16.8 ± 4.2	54.2 ± 16.7	< 0.001	-	-	-

Values are n (%) or mean ± SD, unless otherwise noted

* Based on the mean of the simplified disease activity index at admission and follow-up [9]

SDAI, Simplified disease activity index. DMARDs, disease-modifying anti-rheumatic drugs

**Table 2:** Comparison of complications after total hip arthroplasty in 337 rheumatoid arthritis patients, stratified by mean disease activity*

Complication	Remission (*n* = 10)	Low (*n* = 106)	Moderate (*n* = 118)	High (*n* = 103)	*p*
All complications combined	0	3 (2.8)	17 (14.4)	18 (17.5)	0.003
Dislocation	0	0	8 (6.8)	7 (6.8)	**0.041**
Infection	0	2 (1.9)	4 (3.4)	10 (9.7)	**0.037**
Superficial infection	0	2 (1.9)	2 (1.7)	8 (7.8)	0.052
Periprosthetic joint infection	0	0	2 (1.7)	2 (1.9)	0.541
Periprosthetic fracture	0	1 (0.9)	1 (0.8)	0	0.796
Aseptic loosening	0	0	4 (3.4)	1 (1.0)	0.182

Values are n (%), unless otherwise noted

* Based on the mean of the simplified disease activity index at admission and follow-up

**Table 3:** Logistic regression to identify factors associated with risk of complications in rheumatoid arthritis patients after total hip arthroplasty

Factor	Odds ratio	95% confidence interval	*p*
Mean simplified disease activity index	1.015	1.001–1.029	**0.035**
Disease-modifying anti-rheumatic drugs	1.325	0.587–2.992	0.498
Glucocorticoids	0.674	0.318–1.426	0.302
Rheumatoid arthritis (reference: osteoarthritis)	2.932	1.558–5.520	**< 0.001**

**Table 4:** Comparison of complications after total hip arthroplasty between rheumatoid arthritis patients and osteoarthritis patients

Complication	Rheumatoid arthritis (*N* = 337)	Osteoarthritis patients (*N* = 337)	*p*
All complications combined	38 (11.3)	14 (4.2)	**0.001**
Dislocation	15 (4.5)	6 (1.8)	**0.046**
Infection	16 (4.7)	4 (1.2)	**0.006**
Superficial infection	12 (3.6)	3 (0.9)	**0.019**
Periprosthetic joint infection	4 (1.2)	1 (0.3)	0.178
Periprosthetic fracture	2 (0.6)	1 (0.3)	0.563
Aseptic loosening	5 (1.5)	3 (0.9)	0.477

Values are n (%), unless otherwise noted

When we compared the entire groups of rheumatoid arthritis patients and osteoarthritis patients, we found that rheumatoid arthritis patients showed significantly higher incidence of all complications together, as well as higher rates of dislocation, superficial infection and all types of infection together (Table 4). Logistic regression linked rheumatoid arthritis to significantly higher risk of postoperative complications in general (OR 2.932, 95% CI 1.558–5.520, *p* < 0.001).

## Discussion

Our study appears to be the first to explore possible associations between mean SDAI of rheumatoid arthritis and risk of complications after total hip arthroplasty. It suggests that higher activity is associated with greater risk of postoperative complications in general and of dislocation and superficial infection in particular. One explanation is that more severe systemic inflammation increases risk of postoperative complications, which is supported by our finding that osteoarthritis patients are at significantly lower risk of complications after total hip arthroplasty than rheumatoid arthritis are, even after controlling for numerous clinicodemographic factors.

Our work extends previous studies that have implicated several factors in risk of dislocation after total hip arthroplasty among rheumatoid arthritis patients [13–16]. One factor is female sex: rheumatoid arthritis is more common in women, who tend to have weaker muscles around the hip joint than men. Another factor is less exercise: rheumatoid arthritis patients tend to exercise less because of hip pain, joint deformity and other factors, leading to weak hip abductor muscles and soft tissue relaxation. A third factor is lower body mass index: chronic inflammation tends to reduce it, which may lead surgeons to prefer smaller femoral head components. Our rheumatoid arthritis patients were at significantly higher risk of prosthesis dislocation than the matched group of osteoarthritis patients. How systemic inflammation increases risk of dislocation remains unclear and should be explored in future work.

Our observation of greater risk of postoperative infection with more severe activity of rheumatoid arthritis may reflect that disease-modifying anti-rheumatic drugs, systemic corticosteroids, and biologics increase risk of infection in rheumatoid arthritis patients [17, 18]. In addition, long-term use of glucocorticoids can lead to dermal and epidermal atrophy, increasing risk of skin tears that do not heal properly [19]. Similarly, a meta-analysis revealed that patients with a high disease activity prior to total knee arthroplasty (TKA) still face an elevated risk of flares and are unable to achieve remission levels of activity after TKA. Therefore, long-term drug treatment should be continued to manage their condition effectively.

Although loosening in our study occurred only in patients with rheumatoid arthritis of moderate and high disease activity, there was no statistical difference between the two groups. A previous study linked higher SDAI with greater risk of radiographic loosening after total joint arthroplasty in rheumatoid arthritis patients [5]. Similarly, several studies linked more severe disease activity with worse knee function after total knee arthroplasty in rheumatoid arthritis patients [20–22].

Interestingly, we did not find an association between type of drug therapy and risk of postoperative complications in our rheumatoid arthritis patients. Some have suggested that disease-modifying anti-rheumatic drugs may suppress immune responses and thereby increase risk of postoperative infection [7]. Indeed, infliximab and etanercept have been associated with greater risk of periprosthetic joint infection after total joint arthroplasty [23], and the American College of Rheumatology recommends discontinuing biological therapy for at least one week before such surgery [24]. Our failure to observe a link between type of drug therapy and risk of complications may reflect our insistence on timely discontinuation when screening rheumatoid arthritis patients for enrollment. Future work should focus on identifying disease-modifying anti-rheumatic drugs that do not increase risk of infection after total joint arthroplasty.

Our comparison between rheumatoid arthritis patients and osteoarthritis patients is consistent with a meta-analysis of 23 studies concluding that rheumatoid arthritis is associated with greater rates of revision surgery, hip dislocation, and surgical site infection after total hip arthroplasty [25]. It is also consistent with a study involving nearly 44,000 patients who underwent total hip arthroplasty, in which rheumatoid arthritis patients were at higher risk of revision because of early dislocation [4]; and with three studies showing higher risk of dislocation after total hip arthroplasty or higher risk of periprosthetic joint infection after total joint arthroplasty among rheumatoid arthritis patients [26–28]. On the other hand, one study of a large sample from the US reported significantly lower rates of in-hospital complications after total joint arthroplasty and significantly lower in-hospital mortality after total hip arthroplasty among rheumatoid arthritis patients [29]. This apparent discrepancy may reflect better control of disease activity and regular medical follow-up in that study.

Our findings should be interpreted with caution in light of several limitations. The retrospective study design at a single center may increase risk of recruitment and selection bias. Because the postoperative follow-up time of the patients included in our retrospective study was not exactly the same, and there were missing data at some postoperative follow-up time points, we could not statistically analyze how the disease activity changes after THA. We lacked the data to examine the potential longitudinal influence of disease-modifying anti-rheumatic drugs on risk of postoperative complications. We were unable to match rheumatoid arthritis patients and osteoarthritis patients according to inflammation status for lack of validated indices for osteoarthritis. Our results should be verified and extended in larger samples, preferably across multiple study sites with a prospective design.

## Conclusions

Greater mean SDAI in rheumatoid arthritis patients is associated with higher risk of postoperative complications in general and of dislocation or infection in particular after total hip arthroplasty. This association may reflect the effects of systemic inflammation, since risk of these complications was significantly higher among rheumatoid arthritis patients than a carefully matched sample of osteoarthritis patients. Our results highlight the need to reduce or even eliminate disease activity in rheumatoid arthritis patients before total hip arthroplasty [30].

## Data Availability

No datasets were generated or analysed during the current study.

## Abbreviations

SDAI: Simplified disease activity index
OR: Odds ratio
CI: Confidence interval
